# Overweight/Obesity Prevalence among Under-Five Children and Risk Factors in India: A Cross-Sectional Study Using the National Family Health Survey (2015–2016)

**DOI:** 10.3390/nu14173621

**Published:** 2022-09-01

**Authors:** Jay Saha, Pradip Chouhan, Farooq Ahmed, Tanmoy Ghosh, Sabbir Mondal, Muhammad Shahid, Saireen Fatima, Kun Tang

**Affiliations:** 1 Department of Geography, University of Gour Banga (UGB), Malda 732103, West Bengal, India; 2 Vanke School of Public Health, Tsinghua University, Beijing 100029, China; 3 Department of Anthropology, Quaid-i-Azam University Islamabad 44000, Pakistan; 4 School of Insurance and Economics, University of International Business and Economics (UIBE), Beijing 100029, China; 5 Fazaia Medical College, Air University, Islamabad 44000, Pakistan

**Keywords:** overweight/obesity risk, under-five children, dietary diversity, NFHS, India

## Abstract

The occurrence of overweight and obesity has increased in recent years in India. In this study, we investigate the prevalence and associated risk factors of overweight/obesity among children aged 0–59 months in India. Using data from the 2015–2016 National Family Health Survey-4 (NFHS-4), the research sample included 176,255 children aged 0 to 59 months. Bivariate and multivariate techniques were used to analyze children’s risk factors for overweight/obesity. We identified that the prevalence of overweight/obesity among children aged 0–59 was 2.6% in India. The study findings reveal that factors such as child sex, age, birth weight, birth rank, maternal education, number of children, age at marriage, mother’s BMI, media exposure, social group, and dietary diversity score were most significantly correlated with childhood overweight and obesity in India. Furthermore, we found that male children (ARR: 1.08) aged between 0 and 11 months (ARR: 3.77) with low birth rank (ARR: 1.24), obese (ARR: 1.81) children whose mothers married after the age of 18 (ARR: 1.15), children who belong to a scheduled tribe family (ARR: 1.46), and children who consumed 7–9 food items (ARR: 1.22) were at highest risk of being overweight and obese. However, breastfeeding (ARR: 0.85) and Muslim families (ARR: 0.87) appeared to be protective factors with respect to childhood overweight and obesity in India. Pertinent public health programs, clinical follow-up, and awareness about sedentary lifestyles can help to reduce overweight/obesity risks in children.

## 1. Introduction

Childhood obesity and overweight were initially considered a disease in developed countries with higher per capita income [1]. Overweight is defined as excess body weight relative to height, whereas obesity refers to surplus body fat [2]. According to the World Health Organization (WHO), when body mass index (BMI) is more than 25, the situation is denoted as overweight, and a BMI of more than 30 is considered an obesity condition [3]. The burden of overweight and obesity among children has increased, becoming a global public health concern [4,5]. In developing countries with emerging economies, the increasing trend of overweight and obesity among children poses a significant challenge to the healthcare system [6]. The occurrence of overweight and obesity is higher in developed countries than in developing countries [7]. The prevalence of childhood obesity has increased in developed countries. However, obesity prevalence is also increasing in developing countries [8]. The conditions of overweight and obesity primarily occur due to energy imbalances between calories consumed, calories exhausted, and excessive calorie intake or insufficient physical activity. Childhood overweight/obesity is a precursor to metabolic syndrome, poor physical health, mental disorders, respiratory problems, and glucose intolerance, which can continue into adulthood [9]. Childhood overweight and obesity are determined mainly by insufficient nutrition, physical inactivity, high socioeconomic status, urban residency, traditional beliefs, and marketing of transitional food companies [7,10].

Childhood overweight/obesity is a significant public health concern in the 21st century. At the global level, many middle- and low-income countries are affected by overweight/obesity, particularly in urban areas [8]. According to the WHO, approximately 39 million under-five-year-old children are overweight or obese [3]. Globally, childhood overweight and obesity are associated with more deaths than childhood underweight conditions. Worldwide, overweight/obesity is considered the fifth leading mortality risk factor, now representing a global epidemic. According to Global Burden of Disease 2017, more than four million people die annually as a result of being overweight or obese [4].

On average, 60% of children suffering from overweight/obesity have at least one additional risk factor for cardiovascular diseases, such as hypertension, hyperlipidemia, or hyperinsulinemia [11]. The risk factor for developing abnormal lipid profiles is high among children with overweight/obesity [12,13]. In obese children, high blood pressure is three times greater than in non-obese or normal children [14,15,16]. A cluster of non-communicable diseases and unhealthy lifestyles described as “lifestyle syndrome” or “new world syndrome” has been observed due to the rapid advancement of urbanization and expanding demographic trends [10].

In India, a dual burden has been observed whereby children and adolescents suffer from obesity or overweight on the one hand and malnourishment or underweight on the other hand [17]. The Global Burden of Disease (GBD) report shows that in India, the predominance of overweight children aged 2 to 4 years was 11.5% in 2017 [4]. The tendency of children to be overweight in India increased significantly between 2010 and 2017 and is projected to increase to 17.5% by 2030 [4]. The occurrence of overweight among children in India has increased from 1.6% (2006) to 3.8% (2020) [18]. According to the NFHS report, the prevalence of overweight children under five years of age has increased from 2.1% (2015–2016) to 3.4% (2019–2021).

More than 14.4 million children are obese in India, the second-highest rate globally, behind China [4]. Various studies have suggested possible reasons for the increasing trends of overweight/obesity among children in India. Possible explanations include insufficient physical activity, increased television screen time, urban residency, and family social status [19,20,21,22,23,24]. Further research is required to examine global health issues, such as child obesity/overweight, among young children to protect them from future consequences. Significant differences in overweight/obesity prevalence can be observed according to various factors among under-five children in India. Significant differences have been reported in countrywide representative studies on nutritional status in India according to sociodemographic and household characteristics, as well as dietary characteristics. Therefore, the present research aims to investigate and identify the prevalence of overweight or obesity among Indian children under five years of age, as well as sociodemographic and household risk factors. 

## 2. Materials and Methods

### 2.1. Study Design and Sampling Weights

Data were taken from the fourth round of the NFHS conducted in 2015–2016, a cross-sectional national representative survey to estimate overweight/obesity and its associated factors among children under five years of age. The NFHS 2015–2016 was conducted under the stewardship of India’s Ministry of Health and Family Welfare (MoHFW), with coordination and technical guidance provided by the International Institute of Population Science (IIPS), Mumbai. The countrywide representative sample survey provides comprehensive data on women’s health, child health, and family planning. The NFHS-4 includes data from a population-representative sample of 699,686 women aged 15–49 years and 112,122 men aged 15–54 from 601,509 households. The response rates for women and men were 97% and 92%, respectively. Municipal corporation offices provided a list of 28,586 clusters for this stratified sample (20,509 clusters in rural areas, 8397 clusters in urban areas, and 130 clusters in slums). Sampling weights are necessary for any analysis using the NFHS-4 data to ensure the representativeness of the survey results at the national and domain levels due to the non-proportional allocation of the sample to the different survey domains and urban and rural areas [25]. Because the NFHS-4 sample is a two-stage stratified cluster sample, sampling weights were determined using independent sampling probabilities for each stage and each cluster. The NFHS-4 report of India includes further information on the sampling technique. 

### 2.2. Study Participants

**Figure 1:**
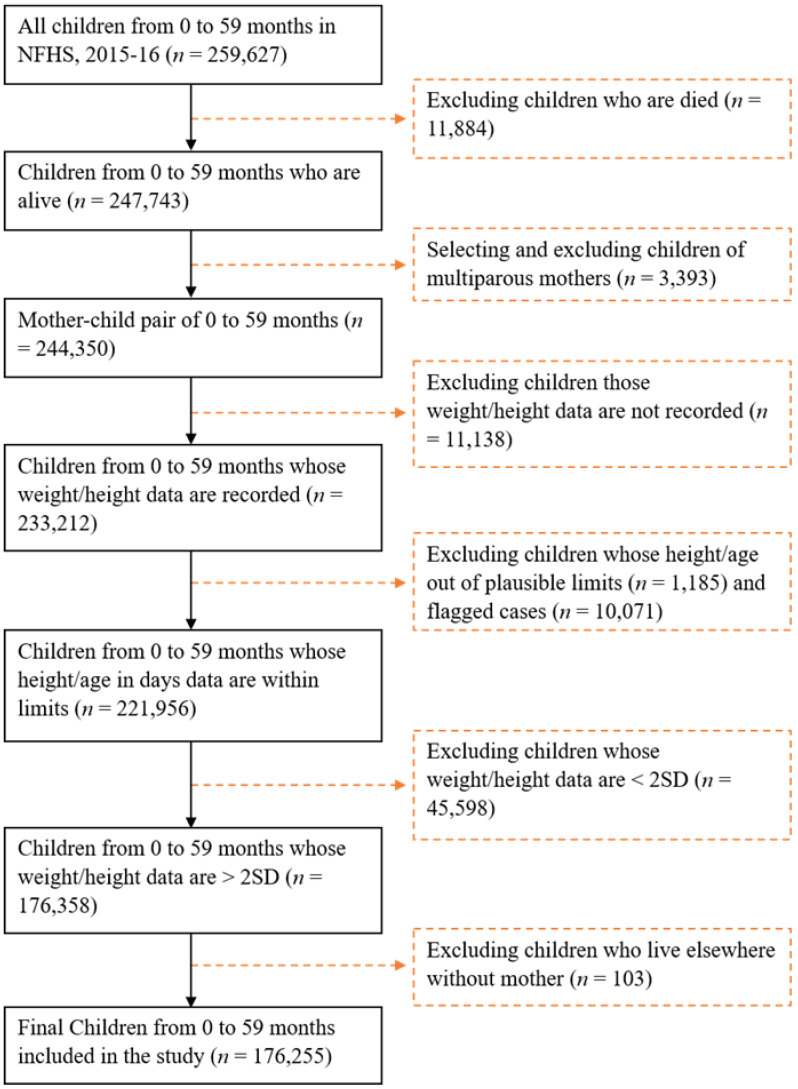
Flow diagram showing children aged 0 to 59 months included in the study for analyses from the 2015–2016 NFHS-4, India.

In the study sample, a total of 259,627 under-five children were born in the last five years (*n* = 259,627), of which 83,372 children were excluded: 11,884 due to death; children of multiparous mothers (*n* = 3393); children whose weight/height data were not recorded (*n* = 11,138); those whose height/age were outside of reasonable limits (*n* = 1185); flagged cases (*n* = 10,071); children whose/height data were <2SD (*n* = 45,598) from the median of the reference population, considered acutely malnourished; and children who lived elsewhere without their mother (*n* = 103) (Figure 1). The height and weight of children between the ages of 0 and 59 months were assessed. The weight of children was determined using a Seca 874 digital scale. A Seca 213 stadiometer was used to measure children’s height between the ages of 24 and 59 months [25]. The recumbent length of children younger than two years old or with a height of less than 85 cm was measured with a Seca 417 infantometer. Children with height-for-age Z scores <−6 SD or >+6 SD, weight-for-age Z scores <−6 SD or >+5 SD, or weight-for-height Z scores <−5 SD or >+5 SD were flagged as having invalid data [25]. Ultimately, 176,255 children aged younger than 59 months were selected for this study (Figure 1). A total of 5130 children were found to be overweight or obese, and these children were included in the study analysis. The remainder of the children (*n* = 171,125) were considered normal-weight children. 

### 2.3. Outcome Characteristics

The study’s outcome variable, child overweight or obesity between 0 and 59 months, assessed sociodemographic and household characteristics based on children’s body mass index Z scores. According to the WHO, a child with a BMI Z score >2SD is considered overweight and obese with a BMI Z score > 3SD [26,27]. In our study, children with a BMI Z score of more than 2 (>2SD) were considered overweight/obese, and those with a BMI Z score in the range of −2 to +2 were classified as normal-weight children. In this study, we dichotomized the binary variable into two categories: normal children, coded as “0”; and overweight/obese children, coded as “1”.

### 2.4. Explanatory Characteristics

We considered independent variables of sociodemographic and household characteristics (child, maternal, and household-level factors). Child-level factors included child sex (male and female), child’s age in months (0–11, 12–23, 24–35, 36–47, and 48–59 months), birth weight (<2.5 kg = low and ≥2.5 kg = normal), currently breastfeeding (no and yes), and birth rank (1, 2, 3, and 4+). Maternal factors included maternal education, comprising four categories (illiterate, primary, secondary, and higher), age at marriage (<18 years and ≥18 years), and the number of children (≥4 and <4). Maternal BMI was categorized into three classes (thin (<18.5 kg/m^2^), normal (18.5–24.9 kg/m^2^), and overweight/obese (>25 kg/m^2^)). Maternal BMI is determined by dividing a woman’s weight in kilograms by their height in square meters (kg/m^2^) [25]. Finally, the household- or community-level factors were divided into the following categories: place of residence (rural and urban), region (categorized into six subdivisions: North: Punjab, Himachal Pradesh, Uttarakhand, Haryana, Chandigarh, Rajasthan, Jammu and Kashmir, and Delhi; Central: Madhya Pradesh, Chhattisgarh, and Uttar Pradesh; East: West Bengal, Bihar, Jharkhand, and Odisha; Northeast: Nagaland, Assam, Manipur, Mizoram, Meghalaya, Tripura, and Sikkim; West: Goa, Dadra and Nagar Haveli, Maharashtra, Daman and Diu, and Gujrat; and South: Andhra Pradesh, Karnataka, Kerala, Telangana, Tamil Nadu, Lakshadweep, and Puducherry) [25], social groups (Scheduled Caste, Scheduled Tribe, Other Backward Classes, and other), religious belief (Hindu, Muslim, and other), and wealth quintile (poorest, poorer, middle, richer, and richest).

#### Explanation of Explanatory Variables

Dietary Diversity Score (DDS): In the NFHS-4, dietary diversity was assessed immediately based on the number of food groups consumed within the last 24 h [25]. Expenditure information was collected on 21 different types of food eaten by children the day before data collection. These foods were initially divided into nine categories: milk or curd, pulses or beans, fruits, eggs, fish, chicken or meat, fried food, dark-green leafy vegetables, and aerated drinks [25]. Based on the data on food consumption (never/rarely, daily, and weekly), a dietary diversity score was calculated and divided into three categories: 3 food items (children who consumed 3 of 9 foods), 4–6 food items (children who consumed 4–6 of 9 foods), and 7–9 food items.

Exposure to media: The frequency of reading newspapers and magazines, watching television, and listening to the radio each week was used as a proxy for media exposure. Based on these three media categories, mothers were classified into three groups: low exposure (uses at least one of these media at least once a week), medium exposure (uses any two of these media at least once a week), and high exposure (uses at least three of these media at least once a week).

Wealth quintile: The household wealth quintile is a score of economic well-being based on housing properties and sustainable product ownership [28]. Based on these scores measured by principal component analysis, household wealth is categorized into five levels: poorest, poorer, middle, richer, and richest. Each level corresponds to 20% of the respondents, ranging from 1 (poorest) to 5 (richest).

### 2.5. Statistical Analyses

Bivariate and multivariate techniques were used to analyze the association between childhood overweight/obesity and sociodemographic, household, and dietary characteristics. The data were also examined using descriptive statistics. We first calculated the proportion of overweight/obese children and that of normal children. The frequency and percentage of the study variable were determined using descriptive statistics as the next step. Pearson’s chi-square tests were used in bivariate analysis to determine the sociodemographic and household characteristics associated with the prevalence of overweight/obesity and the significant level across the independent variables. Binary logistic regression models were used to assess the unadjusted risk ratio (URR) and adjusted risk ratio (ARR) with 95% confidence intervals (C.I.s) between childhood overweight/obesity and sociodemographic and household characteristics. The ARR was controlled for the sex of the child, the child’s age, currently breastfeeding, birth rank, mother’s educational level, age at marriage, mother’s BMI, place of residence, region, social group, religious beliefs, wealth quintile, and dietary diversity score. Data analyses were executed with STATA 12.1 version (StataCorp L.P., Lakeway Drive, College Station, TX, USA).

## 3. Results

### 3.1. Children in Pairs from Different Sociodemographic and Household Characteristics in India

Sociodemographic and household characteristics of children aged 0–59 months and their mothers are depicted in Table 1. Approximately 2.6% of the total sample population was found to have childhood overweight/obesity. The majority of children in the sample were aged between 36 and 47 months. Approximately 85% of children had a normal birth weight (≥2.5 kg), and more than two-thirds of children were currently breastfeeding. The most common birth rank was first or second. Nearly one-third of women had no educational attainment. More than 40% of women were married before the age of 18, and one-third of mothers had more than four children. The body mass index (BMI) of more than 60% of mothers was normal. Only 7% of women were fully exposed to mass media. Most of the children were members of OBCs (46.6%), belonging to the Hindu (78.3%), living in rural areas (71.9%), and from central (27.7%) and eastern (25.8%) regions of India. A large portion of the children were members of the poorest (24.1%) and poorer (21.9%) wealth quintiles. 

### 3.2. Dietary Characteristics of Children in India

Table 2 represents the dietary characteristics of children aged 0–59 months. A significant portion of children consumed milk or curd (42.3%), pulses or beans (44.6%), and dark-green leafy vegetables (46.7%) every day, and nearly all of the children rarely/never ate fruits (58.2%), eggs (59.2%), fish (66.8%), chicken or meat (67.8%), fried food (55.3%), and aerated drinks (78.6%). 

### 3.3. Prevalence of Overweight/Obesity by Sociodemographic and Household Characteristics of Under-Five Children in India

The prevalence of overweight/obesity relative to socioeconomic and household characteristics of under-five children in India is depicted in Table 3. The prevalence of childhood overweight/obesity was found to be significantly higher in the following groups: age of 0–11 months (5.8%), normal birth weight (2.9%), currently breastfeeding (2.7%), and lower birth rank (3%). There was statistically significant variation in childhood overweight or obesity with respect to sex (*p* = 0.006). The rate of obesity/overweight was significantly higher in children with mothers with higher educational qualifications (4.7%), who were married after the age of 18 (3.1%), had fewer children (2.8%), were obese (3.4%), and fully exposed to mass media (4%). The prevalence of overweight/obesity was significantly higher among children residing in urban areas (3.4%), southern regions (3.6%), scheduled tribes (2.9%), belonging to other communities (3.1%), and living in households belonging to the richest wealth quintile (4.1%). 

### 3.4. Prevalence of Overweight/Obesity According to Dietary Characteristics of Under-Five Children in India

Table 4 illustrates the analyses of the causes of overweight/obesity by dietary characteristics of under-five children in India. Children who consumed milk or curd (3.1%), pulses or beans (2.8%), dark-green leafy vegetables (3%), fruits (3.6%), eggs (3.5%), fish (3.3%), chicken or meat (3.6%), fried food (3.3%), and aerated drinks (3.6%) daily were more susceptible to overweight/obesity.

### 3.5. Factors Associated with Childhood Overweight/Obesity in India

Table 5 shows the associations between study variables and childhood overweight/obesity among children aged 0–59 months. Male children had an increased risk of being overweight or obese relative to female children (ARR: 1.08 and 95% CI: 1.02–1.14). Children aged 0–11 months had a 3.7 times higher chance of being overweight/obese than children aged 48–59 months (ARR: 3.77 and 95% CI: 3.41–4.16). Normal birth weight was associated with 1.3 times increased probability of being overweight/obese relative to lower birth weight (LBW) (URR: 1.30 and 95% CI: 1.18–1.43). Children who were currently breastfeeding were at a lower risk of being overweight or obese than non-breastfeeding children (ARR: 0.85 and 95% CI: 0.79–0.92). The risk of overweight or obesity was 1.2 times higher among first-born children (ARR: 1.24 and 95% CI: 1.12–1.38). The unadjusted regression model identified a significant relationship between the educational status of mothers and childhood overweight or obesity. Our analysis also revealed that the likelihood of having an overweight or obese child was increased in mothers with a higher educational level relative to that of illiterate mothers. However, this association was not statistically significant (*p* = 0.1).

Children from families with fewer than four siblings had 1.44 times increased chances of being overweight or obese relative to children from families with four or more siblings. The odds of overweight or obesity were more than one time higher among children whose mothers were married after the age of 18 (ARR: 1.15 and 95% CI: 1.08–1.24) and obese (ARR: 1.81 and 95% CI: 1.62–2.02). The prevalence of overweight or obesity was 1.46 times increased among children whose mothers were fully engaged in mass media. Children living in urban areas in the north-eastern and southern regions among those who belonged to other communities and the richest household quintile had a higher probability of being overweight or obese than other children. However, the adjusted table did not show that this association was statistically significant. In terms of social category, children belonging to a scheduled tribe had 1.4 times increased possibility of being overweight compared to children belonging to a scheduled caste (ARR: 1.46 and 95% CI: 1.31–1.62), and children from families with Muslim religious beliefs had a lower prevalence of overweight/obesity than children from Hindu families (ARR: 0.87 and 95% CI: 0.79–0.96). Children who consumed 7–9 food items had an increased chance of overweight/obesity as compared to children who consumed <3 food items (ARR: 1.22 and 95% CI: 1.12–1.34) (Table 5).

## 4. Discussion

In the present study, we examined the incidence of overweight and obesity among children in India, as well as the contributing factors. According to the survey, 2.6% of Indian children under five years of age were obese or overweight. Compared with other South Asian countries, childhood overweight/obesity was found to be higher in India (2.8%) than in Bangladesh (1.6%) and Nepal (1.4%) and lower than in Maldives (5.4%) and Pakistan (4.9%) [26]. Overweight/obesity among under-five children in India was significantly associated with sex, age, birth weight, birth rank, number of children, age at marriage, mother’s BMI, maternal education, media exposure, social groups, and dietary diversity score.

The study results reveal that male children were more likely to be overweight or obese than female children. This results of the present study are also compatible with evidence from Ethiopia [27], Ghana [29], Nepal [30], Pakistan [31], Cameroon [32], China [33], and Brazil [34]. However, our findings contradicts those of other research showing that female children were more likely to be overweight/obese than male children [35,36] or that sex had no considerable influence on overweight or obesity in children [37]. These contradictory results may be a result of genetic and environmental factors [27], calorie intake, physical activity behaviors [38,39], and social and individual psychology [40].

We found that younger children had an increased probability of being overweight or obese relative to their older counterparts. Previous studies carried out in Indonesia [41], Cameroon [32], and Malaysia [42] showed similar results. This phenomenon could be explained by the fact that young children who are fed formula instead of breast milk might become more overweight or obese than older children [43]. 

A significant association was also revealed between breastfeeding and childhood overweight in the present study. Children who were currently breastfeeding had a lower probability of being overweight or obese than non-breastfeeding children. These findings are consistent with those of previous research from the United States [44], China [45], and Denmark [46]. It is possible that breast milk supplies a moderate amount of calories and nutrients for children, such as sugar, water, protein, and fat [45,47], which can protect against childhood overweight or obesity. 

First-born children were more likely to be overweight or obese compared to children with a higher birth rank (4+) in India. Few researchers have studied the link between birth rank and childhood obesity at an early age. This result is consistent with the results of an investigation in Ethiopia [27,32]. Children with a birth rank of 1–3 were more likely to be overweight/obese than children with a birth rank of >3, according to a cross-sectional examination of 4518 Cameroonian children aged 6–59 months.

A significant determinant of childhood overweight or obesity is maternal education. In India, mothers with higher levels of education had a higher risk of having overweight or obese children. Similar findings were reported in studies in Saudi Arabia [48], China [49], Kazakhstan [50], Nepal [30], and Bangladesh [31]. The following factors may explain this result: children from well-educated households may consume more protein, have higher dietary diversity and increased energy and fat intake, and be more likely to have high levels of lipoprotein in their blood, which might cause them to become overweight or obese [35]. Moreover, educated mothers are more likely to be employed, which could mean that they pay less attention to or observe their children’s physical activity or sitting behavior, such as watching television, less than unemployed mothers, which significantly increases their BMI and obesity [51]. Furthermore, we found that mothers with higher levels of education tended to feed their children different food and consume unnecessary nutrients, which may increase the risk of their children being overweight or obese [52]. 

In the present study, we examined the significant impact that maternal age at marriage had on childhood obesity/overweight in Indian children aged 0–59 months. The odds ratios show that children whose mothers were married after the age of 18 were more likely to be overweight or obese. Children of older mothers or those who married after the age of 18 were more likely to be obese or overweight. [53]. We were not able to clearly interpret this finding; however, a possible explanation is that mothers married at an older age began investing more in their careers, which reduced mother-child interactions and gave them less time to monitor their children’s physical activity, which may lead to their children being overweight or obese. 

Another prominent covariate is the mother’s BMI, which has been strongly associated with childhood overweight or obesity. In the current study, children whose mothers were overweight/obese had a higher risk of becoming overweight or obese than those whose mothers were underweight or thin. Numerous studies have reported maternal BMI as a risk factor for childhood obesity [54,55]. This might be explained by the fact that the evidence of epigenetic processes in the uterus, including DNA methylation and changes in the intestinal microbiome, contributes to obesity in children [56]. Excessive lifestyle exposure (socioeconomic status, food production, marketing, food scarcity, and an obese environment) promote unhealthy behaviors, to which some individuals are susceptible [57,58]. For example, it is possible that mothers were exposed to such complex factors, which contributed to the development of their obesity. In such a case, their children would be more likely to be exposed to the same complex factors, increasing the growth of the uterus and the tendency toward obesity [54].

Our findings are consistent with trends that have been identified in developing countries, but the relations did not remain significant upon multidisciplinary analysis. Children with urban residences were more overweight than rural children in India. However, the adjusted risk ratio was not significant. This result is consistent with those reported in a previous study in Cameroon [32]. Several studies have reported a significant association between childhood overweight and place of residence. Furthermore, overweight children have been reported to more often live in urban areas than rural areas. This finding is in line with those of studies conducted in Peru [59], Poland [60], China [33], and Hawaii [61]. 

The present study also highlights a strong association between region and childhood overweight/obesity. The odds of being overweight were almost 1.07 times higher in north-eastern and southern India than in northern India. However, this association was not statistically significant. Overweight rates were two times higher in northern and eastern India than in other regions [17]. Similarly, a higher prevalence of overweight was observed in north-eastern and southern India than in other regions [62], which could be explained by the higher socioeconomic status of these regions, which may be affected by rapid urbanization and a reduction in the number of urban playgrounds, which may lead to a sedentary lifestyle for children.

The present study also highlights the increased risk of childhood overweight or obesity among scheduled tribe families compared to scheduled caste families. No previous research has examined such an association, possibly ignoring the direct influence of social groups on the development of childhood overweight and obesity. A high accumulation of body fat percentage was observed among Indian tribes [63]. Because most tribes are still untouchable, these outcomes can be partially explained by the lack of healthcare awareness and vaccination confidence [64].

Multivariate analysis has shown a weaker protective effect on children overweight/obese of Muslim religion than Hindu religion in India. A previous study in Cameroon [32] reported that Muslim families might protect their children against being overweight, possibly due to parental choices with respect to a child’s diet that may be influenced by religion. In other words, religion can affect eating habits as a result of adherence to rules that separate religious groups [65]. 

With respect to the relationship between the dietary diversity score and childhood obesity or overweight, we observed a significant gradual increase in the risk of being overweight or obese among children who consumed 7–9 food items daily in India. This result is similar to the results of studies on children in Iran [66], Saudi Arabia [67], and the Dongcheng District of Beijing [68]. The dietary diversity score increased in tandem with the percentage consumption of most food groups, leading to excessive energy intake and obesity [69]. Higher dietary diversity scores were related to increased energy intake, increased consumption of all three components of micronutrients (vitamin A, iodine, and iron), and increased risk of obesity/overweight [70]. Higher dietary diversity scores were also associated with daily consumption of several foods, such as curd or milk, pulses or beans, fish, eggs, fruits, chicken or meat, vegetable, fried food, and aerated drinks, which may lead to increased energy accretion and an increased probability of being overweight or obese among children aged 0–59 months in India.

### Strengths and Weaknesses of the Research

This study has several strengths. The nationally representative data used for respondent selection and the multilevel sampling method reinforce the study results [25], to a large extent, increasing the generalizability of our results for all children aged 0–59 months in India. This study highlights the dietary intake of children and related problems. Despite having its strengths, this study is also subject to some significant limitations. We were unable to determine the causal relationship between the predictive variable and explanatory variables due to the cross-sectional nature of the data, which may have distorted our estimates or resulted in the absence of an association. Another limitation is that this analysis did not include all possible sociodemographic and household variables. The present study explains some sociodemographic and household characteristics of overweight or obesity among Indian children under the age of under-five, but it cannot account for factors related to physical activities or children’s lifestyles. We have superscribed this limitation to address the corresponding bias through a verified data imputation method.

## 5. Conclusions

In the present study, we examined the sociodemographic and household factors associated with overweight or obesity among under-five children in India. Risk factors of overweight include being a male child, having a high birth weight, being aged between 0–23 months, and having a low birth rank, whereas breastfeeding protects against overweight or obesity among children between 0 and 59 months of age. The likelihood of being overweight or obese, having children with more than four siblings, getting married after turning 18, and increased media exposure were also higher in children whose mothers had higher levels of education. This study also indicates a high prevalence of early childhood overweight, with significant disparities between dietary diversity scores and scheduled tribe families in India. However, Muslim families appeared to be a protective factor against childhood overweight/obesity. In terms of preventative strategies, parents should focus on advocacy campaigns to reduce excess weight and obesity and strengthen clinical measures, such as antenatal weight gain monitoring, which could help to counteract overweight or obese children in later life. Further studies, i.e., nutrition education studies on feeding practice and physical activity, should be conducted in higher socioeconomic environments. The government should clinically follow up with children with high birth weight in an effort to prevent later childhood overweight. More studies are needed to investigate other possible risk factors linked to the increase in childhood overweight or obesity in India.

## Figures and Tables

**Table 1:** Sociodemographic and household characteristics (*n* = 176,255).

Characteristics	Frequency (*n*)	Percentage (%)
**Child’s BMI**		
Overweight/obesity	5130	2.6
Normal	171,125	97.4
***Child characteristics***		
**Sex of child**		
Male	90,091	51.4
Female	86,164	48.7
**Child age in months**		
0–11	29,822	16.4
12–23	35,174	20.1
24–35	35,833	20.6
36–47	38,474	22.0
48–59	36,952	21.0
**Birth weight**		
Low (<2.5 kg)	20,056	15.5
Normal (≥2.5 kg)	113,125	84.5
**Currently breastfeeding**		
No	61,722	36.4
Yes	114,533	63.6
**Birth rank**		
1	65,979	38.9
2	54,663	32.3
3	27,968	15.1
4+	27,645	13.7
***Mother Characteristics***		
**Mother’s level of education**		
Illiterate	52,295	29
Primary	25,666	14.1
Secondary	81,248	46.1
Higher	17,046	10.8
**Age at marriage**		
<18 years	65,608	40.7
≥18 years	107,334	59.3
**Number of children**		
≥4	34,496	17.3
<4	141,759	82.7
**Mother’s BMI**		
Thin	38,518	23.2
Normal	110,440	60.7
Overweight/obese	26,673	16.1
**Media exposure**		
Low	113,679	64.5
Medium	49,609	28.4
High	12,967	7.1
***Household/community-level factors***		
**Place of residence**		
Rural	133,644	71.9
Urban	42,611	28.1
**Region**		
North	33,769	13.4
Central	51,135	27.7
East	35,662	25.8
Northeast	27,462	3.8
West	11,187	11.6
South	17,040	17.7
**Social group**		
SC	32,843	22.6
ST	34,349	10.0
OBC	69,164	46.6
Other	31,782	20.8
**Religious belief**		
Hindu	125,550	78.3
Muslim	28,333	16.9
Other	22,259	4.8
**Wealth quintile**		
Poorest	43,257	24.1
Poorer	41,557	21.9
Middle	35,981	20.2
Richer	30,509	18.7
Richest	24,951	15.1

**Table 2:** Dietary characteristics of the sample population (*n* = 176,255).

Characteristics	Frequency (*n*)	Percentage (%)
***Dietary characteristics of children***		
**Food items eaten**		
**Milk or curd**		
Never/rarely	68,494	34.2
Daily	66,574	42.3
Weekly	41,187	23.5
**Pulses or beans**		
Never/rarely	22,819	10.1
Daily	73,236	44.6
Weekly	80,200	45.3
**Dark-green leafy vegetables**		
Never/rarely	26,940	15.0
Daily	84,856	46.7
Weekly	64,459	38.3
**Fruits**		
Never/rarely	106,496	58.2
Daily	16,516	10.5
Weekly	53,243	31.3
**Eggs**		
Never/rarely	111,601	59.2
Daily	5774	4.0
Weekly	58,880	36.8
**Fish**		
Never/rarely	123,552	66.8
Daily	6528	4.8
Weekly	46,175	28.4
**Chicken or meat**		
Never/rarely	122,997	67.8
Daily	2237	1.1
Weekly	51,021	31.1
**Fried food**		
Never/rarely	97,131	55.3
Daily	19,589	8.9
Weekly	59,535	35.8
**Aerated drinks**		
Never/rarely	139,822	78.6
Daily	7258	4.0
Weekly	29,175	17.5

**Table 3:** Prevalence of overweight/obesity by sociodemographic and household characteristics of under-five children in India (*n* = 176,255).

Characteristics	Overweight/Obese Children (Row %)	Normal Children (Row %)	Pearson’s χ2 Value	*p*-Value
***Child characteristics***				
**Sex of child**			7.5	0.006
Male	2.7	97.3		
Female	2.6	97.4		
**Child age in months**			1900	<0.001
0–11	5.8	94.3		
12–23	2.4	97.6		
24–35	1.8	98.2		
36–47	1.8	98.2		
48–59	2	98		
**Birth weight**			28.7	<0.001
Low (<2.5 kg)	2.3	97.7		
Normal (≥2.5 kg)	2.9	97.1		
**Currently breastfeeding**			64.5	<0.001
No	2.5	97.6		
Yes	2.7	97.3		
**Birth rank**			63.4	<0.001
1	3	97.1		
2	2.7	97.4		
3	2.5	97.6		
4+	1.8	98.2		
***Mother characteristics***				
**Mother’s level of education**			213.5	<0.001
Illiterate	2	98		
Primary	2.1	97.9		
Secondary	2.7	97.3		
Higher	4.7	95.3		
**Age at marriage**			177.3	<0.001
<18 years	2	98.1		
≥18 years	3.1	96.9		
**Number of children**			82.3	<0.001
≥4	1.7	98.3		
<4	2.8	97.2		
**Mother’s BMI**			276.9	<0.001
Thin	1.6	98.4		
Normal	2.8	97.2		
Overweight/obese	3.4	96.7		
**Media exposure**			106.2	<0.001
Low	2.3	97.7		
Medium	3.1	96.9		
High	4	96		
***Household/community-level factors***				
**Place of residence**			47.3	<0.001
Rural	2.3	97.7		
Urban	3.4	96.6		
**Region**			417	<0.001
North	3.2	96.8		
Central	2.1	97.9		
East	2.1	97.9		
Northeast	3.3	96.8		
West	2.6	97.4		
South	3.6	96.4		
**Social group**			182.6	<0.001
SC	2.4	97.7		
ST	2.9	97.1		
OBC	2.6	97.4		
Other	2.9	97.1		
**Religious belief**			146.9	<0.001
Hindu	2.6	97.4		
Muslim	2.4	97.7		
Other	3.1	96.9		
**Wealth quintile**			106.2	<0.001
Poorest	1.9	98.1		
Poorer	2.1	97.9		
Middle	2.5	97.5		
Richer	3.1	96.9		
Richest	4.1	95.9		

Note: Data from NFHS-4, India, 2015–2016. Percentages were computed by applying sample weights.

**Table 4:** Prevalence of overweight/obesity according to dietary characteristics of under-five children in India (*n* = 176,255).

Characteristics	Overweight/Obese Children (Row %)	Normal Children (Row %)	Pearson’s χ2 Value	*p*-Value
***Dietary characteristics of children***				
**Food items eaten**				
**Milk or curd**			59.9	<0.001
Never/rarely	2.1	97.9		
Daily	3.1	96.9		
Weekly	2.5	97.5		
**Pulses or beans**			28.7	<0.001
Never/rarely	2.8	97.2		
Daily	2.8	97.2		
Weekly	2.4	97.6		
**Dark-green leafy vegetables**			72.0	<0.001
Never/rarely	2.1	98.0		
Daily	3.0	97.1		
Weekly	2.4	97.6		
**Fruits**			85.3	<0.001
Never/rarely	2.4	97.7		
Daily	3.6	96.4		
Weekly	2.8	97.2		
**Eggs**			76.8	<0.001
Never/rarely	2.4	97.6		
Daily	3.5	96.5		
Weekly	2.8	97.2		
**Fish**			42.1	<0.001
Never/rarely	2.4	97.6		
Daily	3.3	96.7		
Weekly	3.0	97.0		
**Chicken or meat**			57.6	<0.001
Never/rarely	2.5	97.5		
Daily	3.6	96.4		
Weekly	2.9	97.1		
**Fried food**			29.6	<0.001
Never/rarely	2.5	97.5		
Daily	3.3	96.7		
Weekly	2.6	97.4		
**Aerated drinks**			17.0	<0.001
Never/rarely	2.5	97.5		
Daily	3.6	96.4		
Weekly	2.8	97.3		

Note: Data from NFHS-4, India, 2015–2016. Percentages were computed by applying sample weights.

**Table 5:** Factors associated with childhood overweight/obesity in India, NFHS, 2015–2016.

Characteristics	Unadjusted Risk Ratio (URR)— 95% CI	Adjusted Risk Ratio (ARR)— 95% CI
**Sex of child**		
Male	1.08 *** (1.02–1.14)	1.079 ** (1.02–1.14)
Female †	1	1
**Child age in months**		
0–11	3.38 *** (3.12–3.70)	3.77 *** (3.41–4.16)
12–23	1.36 *** (1.24–1.50)	1.47 *** (1.33–1.64)
24–35	0.88 ** (0.80–0.98)	0.89 ** (0.80–0.99)
36–47	0.91 * (0.82–1.01)	0.94 (0.85–1.05)
48–59†	1	1
**Birth weight**		
Low (<2.5 kg) †	1	
Normal (≥2.5 kg)	1.30 *** (1.18–1.43)	
**Currently breastfeeding**		
No †	1	1
Yes	1.28 *** (1.21–1.36)	0.85 *** (0.79–0.92)
**Birth rank**		
1	1.42 *** (1.30–1.56)	1.24 *** (1.12–1.38)
2	1.28 *** (1.16–1.41)	1.12 ** (1.01–1.25)
3	1.21 *** (1.09–1.34)	1.16 *** (1.04–1.30)
4+ †	1	1
**Mother’s level of education**		
Illiterate †	1	1
Primary	1.09 * (0.99–1.20)	0.98 (0.88–1.09)
Secondary	1.29 *** (1.20–1.38)	0.93 (0.85–1.02)
Higher	1.92 *** (1.75–2.11)	1.11 * (0.98–1.26)
**Number of children**		
≥4 †	1	
<4	1.44 *** (1.33–1.55)	
**Age at marriage**		
<18 years †	1	1
≥18 years	1.52 *** (1.43–1.62)	1.15 *** (1.08–1.24)
**Mother’s BMI**		
Thin†	1	1
Normal	1.90 *** (1.75–2.07)	1.70 *** (1.56–1.86)
Overweight/obese	2.19 *** (1.98–2.42)	1.81 *** (1.62–2.02)
**Media exposure**		
Low †	1	
Medium	1.30 *** (1.22–1.38)	
High	1.46 *** (1.33–1.61)	
**Place of residence**		
Rural †	1	1
Urban	1.24 *** (1.17–1.321)	1.06 (0.98–1.14)
**Region**		
North †	1	1
Central	0.61 *** (0.56–0.66)	0.67 *** (0.614–0.737)
East	0.60 *** (0.55–0.66)	0.69 *** (0.616–0.762)
Northeast	1.18 *** (1.09–1.29)	1.07 (0.956–1.202)
West	0.75 *** (0.65–0.84)	0.75 *** (0.649–0.857)
South	1.10 ** (1.00–1.22)	1.07 (0.956–1.194)
**Social group**		
SC †	1	1
ST	1.66 *** (1.48–1.77)	1.46 *** (1.31–1.62)
OBC	1.04 (0.96–1.13)	1.05 (0.96–1.15)
Other	1.32 *** (1.20–1.45)	1.15 *** (1.04–1.27)
**Religious belief**		
Hindu †	1	1
Muslim	0.99 (0.91–1.07)	0.87 *** (0.79–0.96)
Other	1.56 *** (1.45–1.68)	0.94 (0.85–1.05)
**Wealth quintile**		
Poorest †	1	1
Poorer	1.02 (0.93–1.11)	0.84 *** (0.76–0.93)
Middle	1.29 *** (1.18–1.40)	0.98 (0.88–1.09)
Richer	1.38 *** (1.27–1.51)	0.97 (0.87–1.09)
Richest	1.72 *** (1.58–1.88)	1.07 (0.94–1.22)
**Dietary diversity score**		
<3 food items †	1	1
4–6 food items	1.14 *** (1.07–1.22)	1.00 (0.94–1.08)
7–9 food items	1.47 *** (1.36–1.58)	1.22 *** (1.12–1.34)
***Constant***		0.01 ***(0.00952–0.0135)
***Pseudo R2***		0.05
***Log-likelihood***		−20281.044
***Probability χ2***		<0.001
***Mean VIF for ARR model***		1.74

*** if *p* < 0.01, ** if *p* < 0.05, * if *p* < 0.1. CI= confidence interval, † = reference category, VIF = variance inflation factor.

## Data Availability

The general datasets are accessible through the Demographic Health Surveys (DHS) repository. The data used in this work are accessible upon reasonable request from the first author.
